# The Potential Application of Starch and Walnut Shells as Biofillers for Natural Rubber (NR) Composites

**DOI:** 10.3390/ijms23147968

**Published:** 2022-07-19

**Authors:** Anna Sowińska-Baranowska, Magdalena Maciejewska, Paulina Duda

**Affiliations:** Department of Chemistry, Institute of Polymer and Dye Technology, Lodz University of Technology, Stefanowskiego Street 16, 90-537 Lodz, Poland; 225552@edu.p.lodz.pl

**Keywords:** biocomposites, biofillers, natural rubber, starch, walnut shells, ionic liquid, silane

## Abstract

The goal of this study was application of corn starch and ground walnut shells in various amounts by weight as biofillers of natural rubber (NR) biocomposites. Additionally, ionic liquid 1-butyl-3-methylimidazolium chloride (BmiCl) and (3-aminopropyl)-triethoxysilane (APTES) were used to increase the activity of biofillers and to improve the curing characteristics of NR composites. The effect of biofillers used and their modification with aminosilane or ionic liquid on the curing characteristics of NR composites and their functional properties, including crosslink density, mechanical properties in static and dynamic conditions, hardness, thermal stability and resistance to thermo-oxidative aging were investigated. Starch and ground walnut shells were classified as inactive fillers, which can be used alternatively to commercial inactive fillers, e.g., chalk. BmiCl and APTES were successfully used to support the vulcanization and to improve the dispersion of biofillers in NR elastomer matrix. Vulcanizates with starch, especially those containing APTES and BmiCl, exhibited improved tensile properties due to the higher crosslink density and homogenous dispersion of starch, which resulted from BmiCl addition. NR filled with ground walnut shells demonstrated improved resistance to thermo-oxidative aging. It resulted from lignin present in walnut shells, the components of which belong to polyphenols, that have an antioxidant activity.

## 1. Introduction

The global market for the production and industrial applications of biopolymers is constantly developing owing to the high consumer demand. The application of biodegradable and environmentally friendly materials has a positive influence on the right balance of waste management. In recent years, biocomposites are increasingly used in various sectors of the economy, particularly in the rubber industry.

Conventional rubber fillers are organic or inorganic substances, that are introduced to the elastomer matrix to improve mechanical, thermal, chemical, dielectric or processing properties of rubber composites and to reduce production costs by increasing the weight and volume of the final rubber product [1]. The most commonly used fillers include chalk, hydrocarbon fillers such as carbon black, graphite, carbon fibers, and also silicates, i.e., kaolin, talc and many more [2]. For years, carbon black has been the most used reinforcing filler in the rubber compounding industry. However, due to its nonrenewable character and origin from petroleum, as well as the tendency of carbon black particles to agglomerate, the application of carbon black in elastomers is not only harmful to the environment but also consumes much energy [3]. In contrast, using of talc or chalk as fillers does not strengthen elastomer composites. These fillers are inactive and only have the function of increasing the weight of the composition and thus reducing costs. Therefore, it is still needed to use cheap, renewable and environmental-friendly fillers for rubber compounding.

According to European Bioplastics, a plastic material is defined as a bioplastic if it is either biodegradable or biobased or features both properties. Biocomposites are biobased materials and have no harmful effect on the environment; their disposal results in less air pollution and global warming [4]. During decomposition, biocomposites do not release toxic chemicals; instead, degradation products, i.e., biomass, CO_2_, water, methane and other products with low molecular weight, can be absorbed back into the earth [5]. Technological development and awareness of environmental protection mean that biocomposites reinforced with natural fibers play an increasingly important role in many branches of the economy [6,7,8,9]. However, use of biocomposites containing biofillers, e.g., natural fibers, is also associated with some disadvantages, i.e., lower strength and durability than synthetic fibers and high moisture absorption and therefore increased swelling. Such limitations together with other difficulties resulted from incorporation of biofillers are a driving force for scientists in order to overcome them and thus obtain multifunctional, eco-friendly materials in line with the requirements of a developing economy.

Starch is common in nature and can be obtained from cereals (wheat, corn and rice), roots or tubers (potatoes and cassava) and sugar, fruits and vegetables [10]. The starch produced by plant cells is defined as a semicrystalline biopolymer with a general formula (C_6_H_10_O_5_)_n_. It has a granular form and consists mainly of two types of polysaccharides: amylopectin and amylose, with different structures and functions. There are also limited amounts of lipids proteins and minerals [11]. Starch is an important substance for developing sustainable materials due to its low cost, biodegradable properties and renewability. A lot of scientists developed starch-based polymers to protect a petrochemical resources and reduce environmental effect [12,13,14]. However, starch-based materials have some disadvantages, including poor mechanical properties, short term stability and sensitivity to aging. Moreover, direct incorporation of hydrophilic starch into natural rubber (NR) matrix results in deterioration of the mechanical properties of NR composites [12]. Therefore, several approaches have been conducted to improve the compatibility between the starch and NR elastomer, i.e., modification of polymer matrix before mixing with starch [15], application of compatibilizer to improve the interaction between starch and polymer matrix [16] or starch modification before mixing with a polymer [17,18].

In the last decade, ionic liquids (ILs) have gained more interest as solvents for polysaccharides, including starches [19,20]. The solubility of starch in ILs depends on the origin of starch, structure and type of ionic liquid (IL) and the temperature at which dissolution is carried out [21,22]. Among ILs, imidazolium salts provide many essential biophysical applications. Polymers modified with imidazolium ILs show antibacterial activity and improved biodegradability [23]. According to the literature, it is known that 1-butyl-3-methyl-imidazolium chloride (BmiCl) is the best IL to dissolve starch [20,22,24,25]. To our knowledge, there is no information in the literature on the use of starch mixed with BmiCl prior to processing as a biofiller for NR elastomer composites.

Walnut shells are one of the agricultural biomass waste materials sourced from the walnut dry fruit [26]. Walnuts are widely planted in the world. In recent years, walnut planting areas have developed rapidly in China [27]. Walnut consists of 60% kernel (the oily material) and 40% of shell (the hard covering of the walnut). The kernel of walnut has wide applications in many sectors of the economy [26]. It is mainly used as dry fruit for medicine preparation, cosmetics, and several other applications. In contrast, the nutshell often remains unused as a waste. Li et al. [28] reported the chemical composition of walnut shells, comprised of cellulose, hemicellulose, and lignin. Walnut shells are natural cellulosic waste containing almost 36.9% of lignin, 36.1% of hemicelluloses and 17.7% of cellulose [29]. However, the chemical composition of walnut shells may vary depending on the geographic area where they are grown. The shell waste, which contains a large amount of lignocellulose, has no utilization except heating purposes. It can be used to produce diverse forms of energy by pyrolysis, i.e., combustion in domestic applications or burning in open environment. Therefore, walnut shells may be considered as a potential source of chemical feedstock and biofuels [26]. Nevertheless, its usage as a biofiller material would make this waste a valuable by-product. Karaagaç et al. [30] has reported the application of pistachio shells as reinforcing filler in natural rubber/styrene–butadiene (NR/SBR) rubber composites. NR/SBR blends with pistachio shells were characterized by high abrasion resistance. The reinforcing effect of shells or other biowaste was also reported by other authors [31,32]. For example, Barczewski et al. [33] studied chestnut shell waste in poly (lactic acid) (PLA) biocomposites. The chestnut shells significantly increased the storage modulus of the samples, whereas tensile properties of the PLA composites were deteriorated due to the addition of such biofiller. Therefore, it is essential to use various types of additives and compatibilizers to improve the properties of final polymer products containing nut shells.

In order to improve the polymer–filler interactions, and thus the performance of vulcanizates, many modifications are used, including treatment of the filler surface with coupling agent prior to incorporation to the polymer matrix or adding a coupling agent directly to the composite. Moreover, most elastomers are nonpolar, whereas in many cases biofillers are hydrophilic. Silanes are widely known additives, used for improving the interfacial adhesion and compatibility between fillers and elastomers [34]. However, the studies mainly concern the common fillers, i.e., silica [35], carbon black and carbon nanofibers [36]. Several authors have also reported the attempts to improve the compatibility between natural fibers and elastomer by using the compatibilizer or biofiller surface modification [34,37,38,39].

In this work, we applied corn starch and ground walnut shells as biofillers of NR elastomer biocomposites. Studies have been performed to prove that these biofillers can be successfully used as inactive fillers alternative to chalk or talc. Additionally, ionic liquid BmiCl and aminosilane such as (3-aminopropyl)-triethoxysilane (APTES) were applied to enhance the vulcanization process of NR compounds and to improve the compatibility between NR elastomer and biofillers tested and thus the useful properties of NR vulcanizates. Furthermore, the influence of the amount of biofiller used on the properties of NR composites was explored. Biobased materials have been repeatedly reported as not improving the properties of polymer composites [40]; thus, we expected that both biofillers and additives, i.e., BmiCl and APTES, may affect the curing characteristics, mechanical properties and resistance of NR composites to thermo-oxidative aging. However, from the point of view of waste management, it is important to ensure that these biofillers do not deteriorate the most important properties of the polymer composites.

## 2. Results and Discussion

### 2.1. Cure Characteristics and Crosslink Density of NR Composites

The rheometric properties of NR compounds were investigated to determine the effect of the content and the type of biofiller used as well as APTES and BmiCl on the curing parameters. The rheometric measurements were performed at 160 °C and the results are summarized in Table 1.

The minimum rheometric torque (S_min_) is a measure of the viscosity of the uncrosslinked rubber compound. The value of S_min_ for the unfilled NR compound was 0.5 dNm. Considering the measurement error, addition of biofillers and both APTES and BmiCl did not significantly affect the S_min_ value and therefore the viscosity of NR biocomposites (Table 1). This is important for technological reasons, since the viscosity of the uncrosslinked rubber compounds considerably affects their processing, especially by extrusion or injection molding.

The maximum torque (S_max_) depends on the crosslinking degree of the elastomer. It is also affected by the hydrodynamic effect of the filler used. The unfilled NR compound showed S_max_ value of 5.4 dNm. Based on the obtained results, it was concluded that the type of biofiller used had no significant influence on the S_max_ values. NR compounds filled with starch, especially those containing 20 phr of this bioadditive, showed higher S_max_ compared to unfilled NR, which was directly related to the increase in the stiffness of the composition [41]. Moreover, increasing the content of starch in NR composite caused an increase in S_max_ of approximately 1 dNm. Regardless of the biofiller’s amount, NR compounds containing walnut shells exhibited slightly higher S_max_ compared to the unfilled NR and similar to that of the rubber compound filled with 10 phr of starch. The highest S_max_ was observed for NR compounds containing APTES and BmiCl, especially those filled with starch. In our previous work [42] we also observed the beneficial effect of imidazolium ionic liquids on the S_max_ of NR compounds. The positive influence of APTES and BmiCl on the S_max_ could result from both the improved compatibility of the biofillers used with NR matrix and the increased crosslinking degree of the elastomer.

The rheometric torque increase (∆S) results from the increase in the stiffness of rubber composite during rheometric measurement; thus, it can be considered as a measure of the crosslinking degree of elastomer. In the case of rubber compounds containing fillers, ∆S is also affected by the hydrodynamic effect of the filler used. Regarding the examined NR compounds, the influence of the filler, APTES and BmiCl on ∆S fully correlated with their effect on the values of S_max_. Consequently, rubber compounds containing biofillers, especially starch, exhibited higher ∆S compared to the unfilled benchmark. ∆S of the starch-filled NR compounds increased with the content of the filler, whereas the number of walnut shells did not significantly affect the values of ∆S. The most pronounced improvement of ∆S was observed for NR compounds containing the ionic liquid BmiCl. NR compounds with BmiCl were demonstrated to be approximately 3.4 dNm (for starch) and 2.4 dNm (for walnut shells) higher ∆S compared to the unfilled benchmark. It was probably due to the improved polymer–biofiller compatibility after introducing BmiCl and beneficial influence of BmiCl on the crosslinking degree of elastomer. It should be noticed that NR compounds containing APTES were also characterized by the higher ∆S compared to the unfilled benchmark and rubber compounds without this additive. However, the improvement of ∆S resulting from APTES addition was slightly smaller as compared to the BmiCl.

The scorch time (t_02_) defines the safety of the processing of rubber composites at a certain temperature. The longer the t_02_, the more secure the processing; thus, the rubber compound can be processed without the risk of scorching. The unfilled rubber compound exhibited a t_02_ of 2 min, whereas the scorch time for the NR compounds filled with starch and walnut shells was extended by approximately 1 min, thus resulting in the slightly improved safety of the processing. In contrast, APTES and BmiCl reduced the t_02_ of the filled NR compounds to 1 min.

The optimal vulcanization time (t_90_) is a crucial parameter from a technological and economic point of view. In the industry, shorter vulcanization times are more desirable because of the reduction of the total cost of final rubber products by reducing the cost of the rubber compound’s vulcanization. The unfilled rubber compound exhibited the t_90_ of 7 min. Rubber compounds filled with starch or walnut shells demonstrated t_90_ to be approximately 2–3 min shorter compared to the unfilled benchmark. Therefore, the accelerating effect of starch and walnut shells on vulcanization was observed. The accelerating influence of starch on the vulcanization of NR was reported by Riyajan et al. [43] and Alwaan [44], who postulated that the interaction between the hydroxyl groups of starch and the double bond of natural rubber enhanced the crosslinking reactions and boosted the vulcanization of NR. Similarly to starch, the walnut shells are composed mainly of polysaccharides and contain some minerals, such as Ca, Mg and Zn, which can support the vulcanization [45]. Regardless of the type of biofiller used, APTES and BmiCl shortened t_90_ by about 4 min as compared to the benchmark. Hence, APTES and BmiCl shortened both t_02_ and t_90_ of rubber compounds, which proves their accelerating effect on vulcanization. The beneficial influence of silane on the vulcanization was confirmed by other researchers [46], whereas the accelerating effect of ionic liquids on the crosslinking reaction was widely reported in the literature [42,47,48,49]. Ionic liquids are considered to be the catalysts of interphase reactions [50]. Since the crosslinking reactions take place at the interface between the elastomer and the curatives, ionic liquids can catalyze crosslinking reactions and thus increase their rate and the crosslinking degree of the elastomer. Moreover, ionic liquids have been reported to improve the dispersion of the components of the curing system in the elastomer matrix, thereby increasing the contact between them and the elastomer chains, which may boost the vulcanization [42,47]. In contrast, fillers have a tendency to partially adsorb the curatives, especially some accelerators. It has been shown that ionic liquids can preferentially adsorb on the surface of the filler, reducing its ability to adsorb curatives and thus increasing vulcanization rate and efficiency [48,49].

The crosslink density (ν_t_) of NR vulcanizates filled with both starch and walnut shells was greater compared to the unfilled sample (Table 1). Moreover, the highest ν_t_ exhibited by vulcanizates filled with starch, which confirmed the beneficial effect of this biofiller on the crosslinking of NR compounds. As expected, APTES and BmiCl increased the ν_t_ of NR vulcanizates, especially those filled with starch, which was probably due to their positive effect on the dispersion of biofillers in the elastomer matrix and vulcanization of NR compounds. Moreover, if the filler has the ability to adsorb the curatives on the surface, the silane and the ionic liquid may preferentially be adsorbed on the surface of the filler, reducing its ability to adsorb the curatives, thus improving the crosslinking efficiency and consequently the crosslinks density of the vulcanizates [42,48,51]. Most importantly, application of starch and walnut shells as biofillers of NR composites instead of other inactive fillers, e.g., talc, could be reasonable, since talc deteriorates the crosslink density of vulcanizates and therefore their mechanical properties [52]. Application of starch and walnut shells allowed us to obtain similar or even better cure characteristics and crosslink densities as compared to the results obtained by Masłowski et al. for NR compounds filled with chalk or talc [52]. Moreover, aminosilane and ionic liquid BmiCl could act as vulcanization supporting agents, and therefore the efficiency of NR vulcanization could be improved.

**Figure 1:**
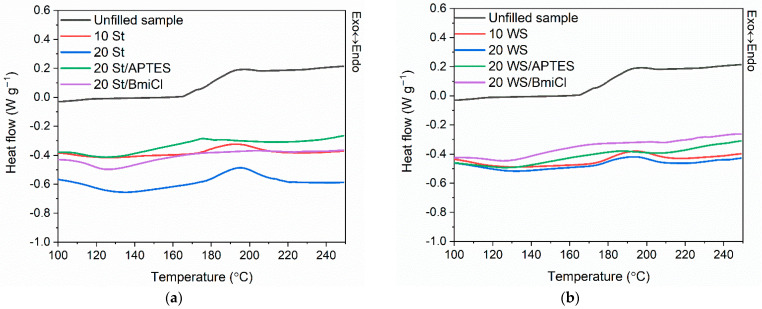
Differential scanning calorimetry (DSC) curves of the NR compounds filled with: (**a**) starch; (**b**) walnut shells.

In the next step of the research, differential scanning calorimetry (DSC) was employed to explore the influence of biofillers and other additives, i.e., silane and BmiCl, on the crosslinking temperature and the enthalpy of crosslinking reactions. The influence of biofillers APTES and BmiCl on the glass transition temperature (T_g_) of the NR elastomer was determined as well. The results of DSC analysis are summarized in Table 2. DSC curves of NR composites filled with starch and walnut shells are plotted in Figure 1.

Analyzing the differential scanning calorimetry (DSC) results, the change in the heat capacity (∆C_p_) presented in Table 2 resulted from the glass transition of NR. The T_g_ of NR determined for the unfilled benchmark was approximately −62 °C, so typical for NR [42,51]. The addition of biofillers, as well as silane and BmiCl, did not affect the T_g_ of the elastomer.

By analyzing the DSC plots (Figure 1), the crosslinking of NR compounds was observed as a one-step exothermic process, in the temperature range dependent on the composition of the rubber compounds, mainly on the presence of silane and ionic liquid. Crosslinking of the unfilled rubber compound occurred in the temperature range of 167–227 °C with an enthalpy (∆H) of approximately 15.6 J/g. The addition and the amount of biofillers did not significantly influence the range of the crosslinking temperature of NR compounds, whereas the enthalpy of crosslinking (∆H) was reduced as compared to unfilled benchmark. In the case of both biofillers, the addition of silane and BmiCl significantly influenced the temperature of crosslinking. The onset temperature of crosslinking was reduced by approximately 37–34 °C compared to other NR compounds, including the unfilled one. Moreover, both APTES and BmiCl increased the energetic effect of crosslinking compared to rubber compounds without these additives. It confirmed the beneficial effect of silane and BmiCl on the crosslinking reactions of NR filled with starch and walnut shells. Therefore, they can be used as substances promoting crosslinking reactions. APTES and BmiCl had more significant influence on the vulcanization of starch-filled NR as compared to rubber compounds containing walnut shells. It was also confirmed by the higher crosslinking density of the vulcanizates containing starch. In the case of BmiCl, it probably resulted from the ability of BmiCl to dissolve the starch, and thus the compatibility of starch with NR elastomer was enhanced compared to rubber composites without BmiCl [18,53,54]. In contrast, the positive effect of APTES on the crosslinking reactions could result from the possible combining of organo-silanes with starch and forming C-Si-O-connects with the hydrogen bonding. Moreover, silane coupling agents improve fillers dispersion in the elastomer matrix and flow behavior, thus affecting the heat transfer inside the elastomer matrix [55,56]. However, it should be confirmed with further research.

### 2.2. Dispersion of Biofillers and Curatives in NR Composites

**Figure 2:**
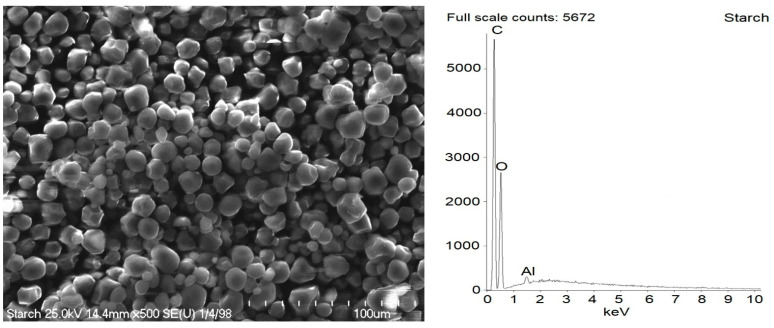
Scanning electron microscopy (SEM) with energy-dispersive X-ray spectroscopy (EDS) analysis for pure starch.

**Figure 3:**
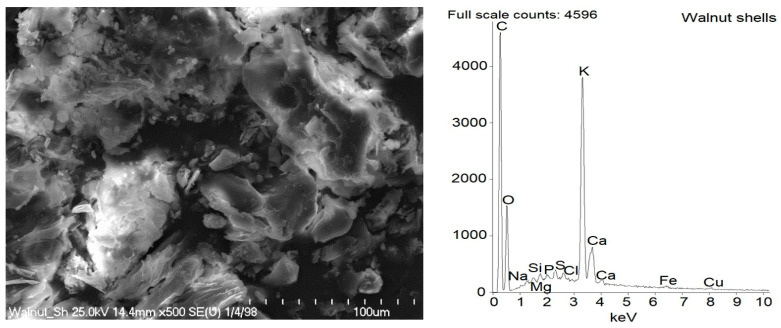
SEM with EDS analysis for pure ground walnut shells.

**Figure 4:**
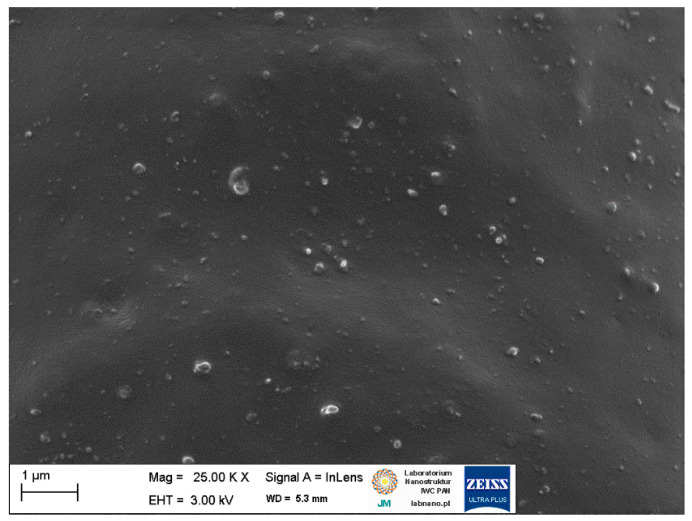
Scanning electron microscopy (SEM) image of the unfilled NR vulcanizate.

**Figure 5:**
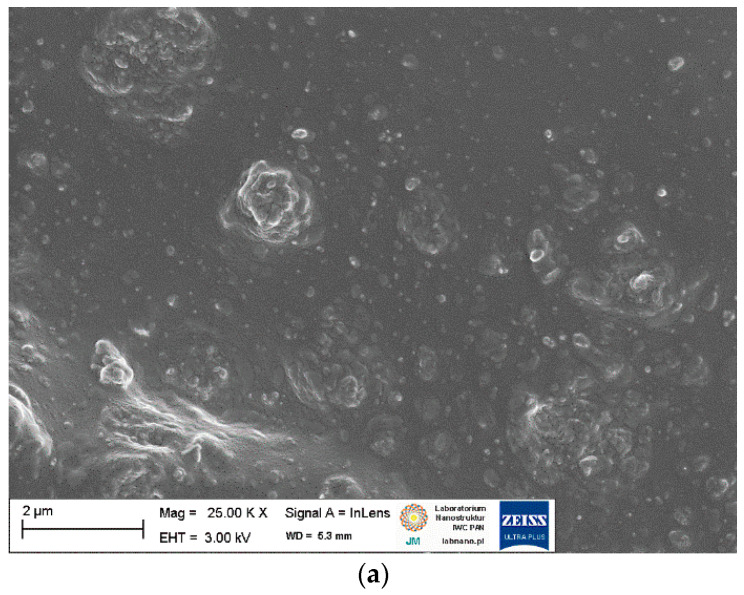

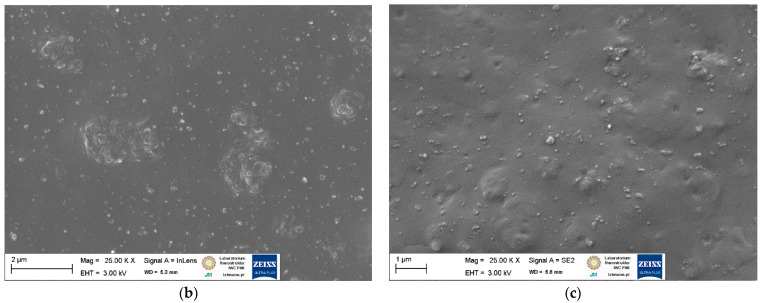
SEM images of NR vulcanizates filled with starch: (**a**) 20 St; (**b**) 20 St/APTES; (**c**) 20 St/BmiCl.

**Figure 6:**
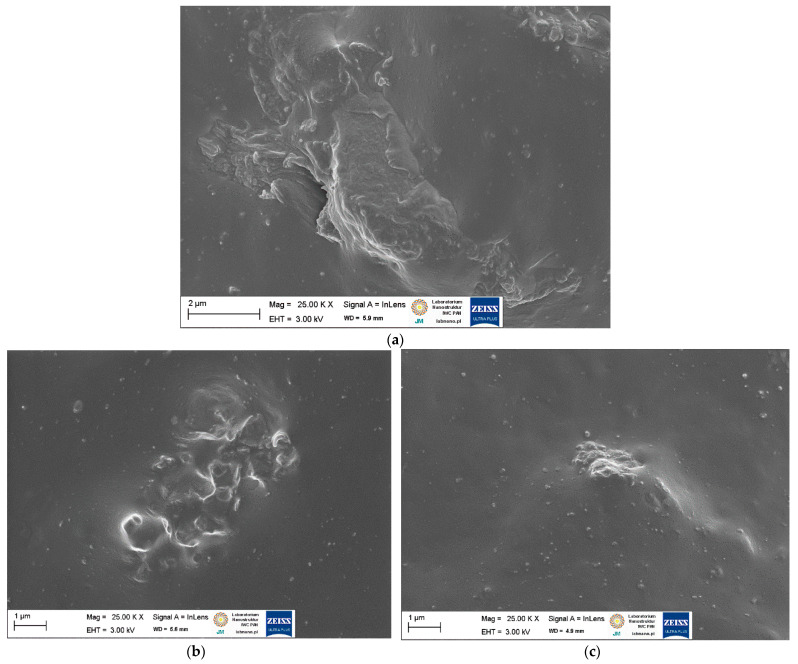
SEM images of NR vulcanizates filled with walnut shells: (**a**) 20 WS; (**b**) 20 WS/APTES; (**c**) 20 WS/BmiCl.

SEM images of pure starch and ground walnut shells powders were taken to study the morphology and size of biofiller particles. Additionally, EDS analysis was employed to estimate the chemical composition of starch and ground walnut shells. Obtained results for pure biofillers are shown in Figure 2 and Figure 3 and Table 3. In elastomer technology the homogenous dispersion of the filler and curatives and distribution of their particles in elastomer matrix is a very important aspect. The primary particles of the filler can form aggregates and agglomerates because of binding by physicochemical forces. The spatial network of the filler in the elastomer matrix depends on the content of filler, its activity and interaction with curatives [1]. The structure, type of filler particles and the degree of filling determine the intensity of interfacial interactions and the morphology of the system and hence influence the functional properties of the composite [2]. Moreover, the interaction between the elastomer matrix and curatives should be maximized to improve the crosslinking efficiency and consequently the functional properties of final products. It has also been proven that some additives can act as plasticizers or solvents for fillers, thus improving their dispersion in elastomer [12,14,17]. Therefore, the impact of APTES and BmiCl on the dispersion of biofillers and curatives was investigated using scanning electron microscopy (SEM). Results obtained for the unfilled benchmark are presented in Figure 4, whereas SEM images for NR vulcanizates filled with starch and walnut shells are given in Figure 5 and Figure 6, respectively.

SEM images showed that primary particles of pure starch exhibited spherical morphology and average size of approximately 10 µm (Figure 2). Starch is a plant polysaccharide. Thus, as expected, C and O elements were present in the EDS spectra, and the content of each was of approximately 50 wt.%. The small Al band in the EDS spectra of pure starch resulted from the contamination. Aluminum is the third most abundant element in the earth’s crust. Moreover, it is also present in soils.

In the SEM image of pure ground walnut shells (Figure 3), the morphology of their particles were complex and irregular, which resulted from the mechanical grinding process. The particles were very heterogeneous in size. Both small particles of about 10 µm and large particles of several tens of microns can be seen. Regarding the chemical composition of the ground walnut shells, C and O bands were present in EDS spectra, and the content of these elements was estimated to be 46 wt.% for C and 43 wt.% for O, respectively (Table 3). It was due to the polysaccharides, i.e., cellulose, hemicellulose and lignin, which contain a large number of carbon and oxygen atoms in their structure. Additionally, alkali metal and alkali earth metal, especially K and Ca, were detected, which could be an important factor to provide better scorch safety. A similar composition of ground walnut shells was described by Chundawat et al. [45].

SEM analysis of the unfilled NR vulcanizate confirmed the homogeneity of the obtained elastomer network and good compatibility between the NR elastomer and the curing system. Small particles, much less than 1 µm in size, were homogeneously distributed in the elastomer matrix (Figure 4). Regarding NR composites containing biofillers, SEM images for 20 phr of the biofiller are presented in Figure 5 and Figure 6, as we did not observe significant differences in the dispersion of the 10 phr and 20 phr of the starch and walnut shells in NR composites.

By analyzing the SEM image (Figure 5a) of the NR vulcanizate containing 20 phr of starch, it was seen that the solid particles were not homogeneously dispersed in the NR elastomer matrix. Since a homogeneous dispersion of the curatives was observed for the unfilled vulcanizate, we concluded that the agglomerates with a size of a few micrometers consisted of starch particles. However, the starch agglomerates seem to be surrounded by an elastomeric film that penetrates between the starch particles. SEM image given in Figure 6b shows slightly more uniform dispersion of the starch particles in the NR elastomer containing APTES as compared to vulcanizate without silane. In contrast, BmiCl significantly improved the dispersion of starch in the NR matrix. In the case of the BmiCl-containing vulcanizate, small aggregates of particles with a size below 1 µm were quite homogeneously dispersed and well wetted by the elastomer matrix (Figure 5c). Moreover, the 20 St/BmiCl vulcanizate showed the dispersion of particles similar to the unfilled vulcanizate, and no agglomeration was observed.

In the SEM image presented in Figure 6a, the particles of walnut shells were not homogeneously dispersed in the NR matrix and formed large agglomerates with a size of several micrometers. However, these agglomerates were surrounded by an elastomer film similarly to starch. Addition of APTES caused a slight improvement of the walnut shells dispersion in NR reducing the size of the agglomerates compared to the vulcanizate without silane (Figure 6b). Similarly to starch, the ionic liquid BmiCl significantly improved the dispersion of walnut shells in the NR matrix. In the case of the 20 WS/BmiCl vulcanizate, small agglomerates of particles with a size of approximately 1–2 µm were homogeneously dispersed and well wetted by the elastomer matrix (Figure 6c). Thus, the beneficial influence of ionic liquids on the dispersion of components of elastomer composites was proven, which has been confirmed also in our previous work [57,58]. The beneficial effect of BmiCl on starch and walnut shells dispersion may result from the ability of BmiCl for interaction with polysaccharides through hydrogen bonding, which are likely to reduce the interactions between biofiller particles to prevent agglomeration [59]. Moreover, ionic liquids such as BmiCl were reported to be used as coupling agents, since they can interact directly with both elastomer matrices and fillers [60] or as compatibilizers between hydrophilic fillers and hydrophobic polymers [61].

### 2.3. Tensile Properties and Hardness of NR Composites Filled with Biofillers

The performance of vulcanizates depend on the type and amount of the filler, its structure and characteristic features. No less important is the presence of other additives, which affect the dispersion of the filler in the elastomer matrix and its activity. Therefore, the influence of biofillers, APTES and BmiCl on tensile properties and hardness of NR vulcanizates was studied. The results are presented in Table 4.

Analyzing the data collected in Table 4, the addition of starch and walnut shells did not significantly affect the stress at a relative elongation of 300% (SE_300_) compared to the unfilled vulcanizate. In contrast, vulcanizates containing APTES and BmiCl exhibited slightly higher SE_300_ compared to the unfilled benchmark and vulcanizates without these additives. It resulted from the higher crosslink density of these vulcanizates compared to other samples. It is commonly known that SE_300_ depends strongly on the crosslink density of vulcanizates and increases when the crosslink density increases [62].

Regarding the tensile strength (TS) and elongation at break (EB) of vulcanizates, the unfilled benchmark reached the TS value of 10.4 MPa and EB of approximately 820%. Vulcanizates filled with starch showed slightly improved TS compared to the unfilled benchmark. The amount of starch had no considerable influence on the TS of the vulcanizates. The starch-filled vulcanizate without BmiCl and APTES exhibited approximately 1.3–1.7 MPa higher TS than the unfilled sample, whereas TS of the vulcanizates containing BmiCl and APTES was approximately 1 MPa higher than that of the unfilled NR composite (11.0 MPa for APTES and 11.5 MPa for BmiCl, respectively). Regardless of their amount, application of walnut shells did not significantly affect the TS of vulcanizates compared to the unfilled benchmark. APTES and BmiCl did not improve the TS of the NR vulcanizates containing walnut shells.

The elongation at break (EB) is a parameter strongly dependent on the crosslink density and filler’s addition. Introducing the biofillers, APTES and BmiCl to NR composites reduced the EB by approximately 200–300% as compared to the unfilled benchmark. Moreover, vulcanizates containing BmiCl showed the lowest EB of all tested vulcanizates (507% for starch and 588% for walnut shells, respectively). This resulted from the highest crosslink density of these vulcanizates.

It is known that the hardness is directly proportional to the crosslink density of the vulcanizates and depends on the addition and activity of the filler. Considering the examined vulcanizates, the type of biofiller slightly affected their hardness. As expected, the unfilled benchmark exhibited the lowest hardness of 31 Shore A. Introduction of starch and walnut shells slightly enhanced the hardness of NR vulcanizates compared to the unfilled sample (by 1–3 Shore A). Addition of APTES and BmiCl caused a further increase in the hardness of NR vulcanizates filled with starch, which was due to the higher crosslink density of those vulcanizates as compared to vulcanizates without these additives. In contrast, APTES and BmiCl did not considerably influence the hardness of NR vulcanizates filled with walnut shells.

Taking into account the effect of starch and walnut shells on the tensile properties and hardness of NR vulcanizates, these biofillers can be classified as inactive fillers, which can be used alternatively to commercial inactive fillers, e.g., chalk and talc [52]. Applying starch and walnut shells as inactive biofillers of NR composites seems to be a good way to manage plant waste from the food industry. Starch can be obtained from potato tubers or potato peelings. Walnut shell waste is sufficient to dry and grind to obtain a powder that can be incorporated into the rubber compound. Importantly, during the preparation of the NR compounds, no difficulties were noticed when incorporating the ground walnut shells into the rubber.

### 2.4. Dynamic Mechanical Properties of NR Composites Filled with Biofillers

**Figure 7:**
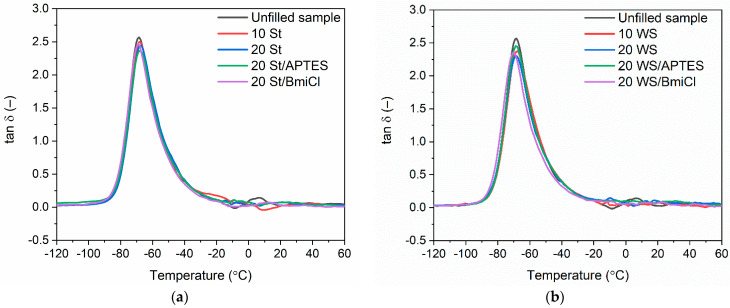
Loss factor (tan δ) curves versus temperature for NR vulcanizates filled with: (**a**) starch; (**b**) walnut shells.

Dynamic mechanical analysis (DMA) was employed to study the influence of biofillers, APTES and BmiCl on the viscoelastic properties of NR composites and their ability to dampen vibrations. The measurements were performed as a function of temperature. The results are summarized in Table 5, whereas the DMA curves of NR vulcanizates filled with starch and walnut shells are plotted in Figure 7.

The DMA curves of NR vulcanizates filled with starch and walnut shells plotted in Figure 7 showed the glass transition of NR elastomer, which occurred in the temperature range of −70 °C to −68 °C, as confirmed by the peak of the mechanical loss factor (tan δ) on the DMA curves. The temperature of the maximum of tan δ peak corresponds to the glass transition temperature (T_g_) of NR elastomer determined in dynamic conditions (oscillating stretching). The T_g_ of NR determined by DMA for the unfilled sample was −68 °C, whereas the T_g_ for the vulcanizates filled with starch and walnut shells was approximately 1–2 °C lower. Therefore, the type and the content of biofiller as well as the additives used did not considerably affect the T_g_ of NR composites, which was within the measurement error.

Mechanical loss factor (tan δ) is a measure of the energy dissipated as heat by the elastomer network during each deformation cycle. By definition, tan δ is the ratio of the loss and the storage moduli (E”/E’) and corresponds to the material’s ability to dampen vibrations [63,64]. As expected, the highest value of tan δ at T_g_ (Table 5) was exhibited by the unfilled vulcanizate. It was approximately 2.6. It resulted from the high flexibility and thus the high mobility of the elastomer chains in the unfilled elastomer network. Addition of biofillers, APTES and BmiCl did not significantly affect the tan δ in T_g_. Considering the measurement error, the values of tan δ were slightly lower compared to that of the unfilled benchmark. Regarding the results obtained for NR containing biofillers used, walnut shells and starch did not significantly affect both the T_g_ and the values of tan at T_g_, so these biofillers had no considerable influence on the mobility of elastomer chains, which is characteristic for inactive fillers [65,66].

Concerning the tan δ at 25 °C and 50 °C, i.e., in the rubbery elastic region (Table 5), addition of the biofillers, APTES and BmiCl did not considerably affect this parameter as compared to the unfilled vulcanizate. The obtained values of the tan δ in the rubbery elastic region showed that NR vulcanizates filled with starch or walnut shells and containing APTES and BmiCl exhibited a good capacity to dampen vibrations and had stable dynamic properties.

### 2.5. Thermo-Oxidative Aging Resistance of NR Composites Filled with Biofillers

**Figure 8:**
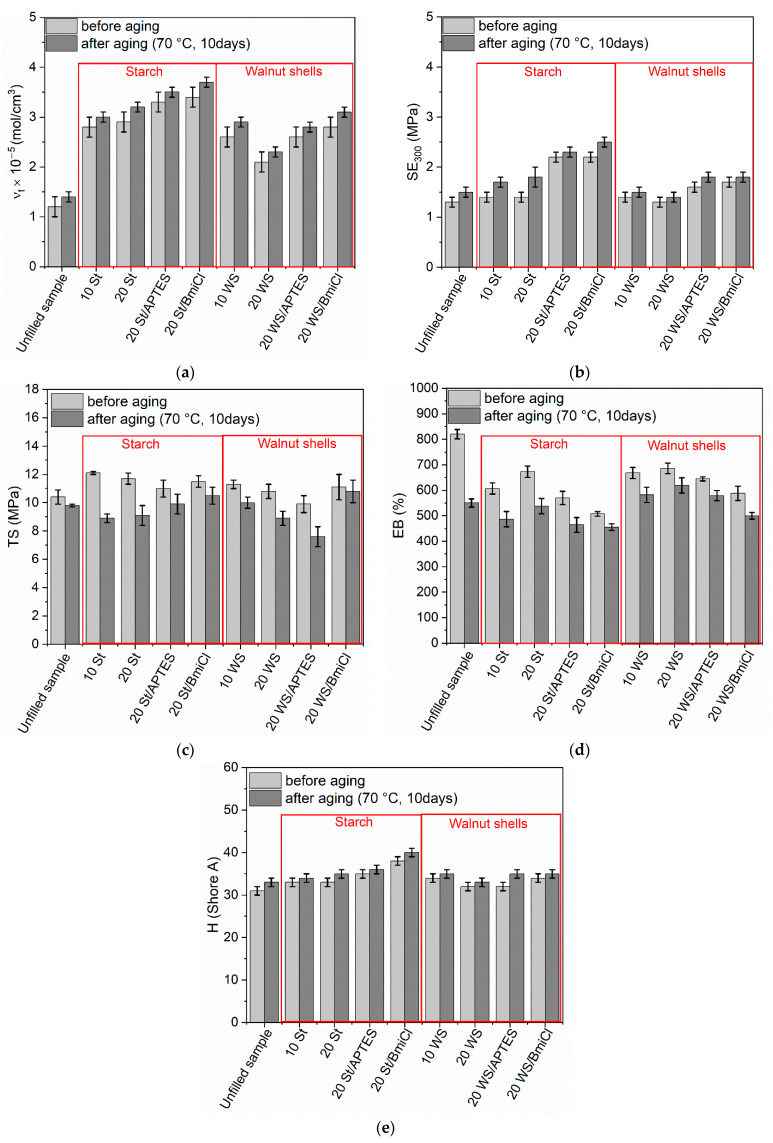
Effect of prolonged thermo-oxidation on the performance and crosslink density of NR composites filled with starch and walnut shells: (**a**) crosslink density; (**b**) stress at 300% relative elongation; (**c**) tensile strength; (**d**) elongation at break; (**e**) hardness.

It is known that NR composites have poor resistance to thermo-oxidative aging as compared to most synthetic rubbers [67]. It has also been proven that some additives such as ILs or silanes can significantly affect the resistance of vulcanizates to the aging process [42,68,69]. Hence, the influence of biofillers, APTES and BmiCl on the resistance of NR vulcanizates to thermo-oxidative aging was studied in the next step of the research. The impact of prolonged thermo-oxidation on the properties of NR composites is grafted in Figure 8.

As expected, vulcanizates containing starch and walnut shells exhibited higher crosslink densities after prolonged exposure to 70 °C (Figure 8a). A similar effect of thermo-oxidation on the crosslink density was achieved for the unfilled benchmark. Increase in the crosslink density of NR vulcanizates due to thermo-oxidation was confirmed by other researchers [70,71]. Choi et al. [72] reported that the changes in crosslink densities due to prolonged exposure to high temperatures resulted from dissociation of the existing sulfur crosslinks in the elastomer network and the formation of new crosslinks by free sulfur.

Since SE_300_ depends on the crosslink density, NR vulcanizates demonstrated higher SE_300_ upon thermo-oxidation as compared to the nonaged samples (Figure 8b).

Thermo-oxidation reduced the tensile strength of the unfilled vulcanizate by approximately 0.6 MPa (Figure 8c). Moreover, thermo-oxidative aging deteriorated the TS of NR vulcanizates filled with starch and walnut shells. Similar influence of thermo-oxidative aging on the TS was achieved for the vulcanizates containing APTES and BmiCl. As mentioned earlier, prolonged exposure to elevated temperatures caused a further crosslinking of NR, resulting in the vulcanizates being over-crosslinked. It is commonly known that over-crosslinked elastomers become brittle and thus show poor resistant to mechanical stress [73].

The unfilled NR vulcanizate exhibited approximately 270% lower EB (Figure 8d) as compared to nonaged material, which was due to the increase in the crosslink density upon the thermo-oxidation. Vulcanizates containing starch and walnut shells were also characterized by lower EB (Figure 8d) compared to nonaged samples. However, the reduction of EB due to aging of vulcanizates containing biofillers was lower compared to the unfilled benchmark.

Thermo-oxidative aging did not significantly affect the hardness of vulcanizates, only by approximately 1–2 Shore A higher compared to that of nonaged samples (Figure 8e).

Having examined the effect of thermo-oxidative aging on the tensile properties of NR vulcanizates containing biofillers, we then determined their aging factor (A_f_). It was calculated using the changes in the TS and EB of vulcanizates resulted from the aging process. The results are summarized in Table 6.

The unfilled NR vulcanizate showed poor resistance to thermo-oxidative degradation processes (Table 6), since the A_f_ reached a value below 1 (A_f_ = 0.6). It resulted from significant negative changes in the TS and EB of the vulcanizates due to the aging process. NR elastomer contains double bonds, which are an active sites for oxidation reactions, leading to the polymer degradation [6].

The application of biofillers did not significantly affect the resistance of NR vulcanizates to thermo-oxidative aging. The A_f_ of the starch-filled composites was approximately 0.6, so the same as for the unfilled sample. Thus, the vulcanizates filled with starch were susceptible to thermo-oxidative aging similarly to the unfilled NR. Vulcanizates filled with walnut shells exhibited better resistance to prolonged thermo-oxidation compared to the unfilled benchmark and vulcanizates containing starch (A_f_ of approximately 0.8). It is known that lignin can act as stabilizer in reactions induced by oxygen and its radical species due to the presence of hindered phenolic groups, thus improving the resistance of polymers to thermo-oxidation [74,75]. The amount of biofiller used had no effect on the resistance to thermo-oxidative aging. Introduction of BmiCl slightly increased A_f_ of the starch-filled vulcanizates. The beneficial effect of ionic liquids on the aging resistance of different elastomers has already been reported by various researchers [42,56,76].

### 2.6. Thermal Stability of NR Composites Filled with Biofillers

Thermal stability of elastomer composites strongly depends on the type and the amount of filler used, especially when biofillers are applied, which contain organic compounds. No less important are other organic additives, i.e., silane and ionic liquid, which decompose when heated to high temperatures.

**Figure 9:**
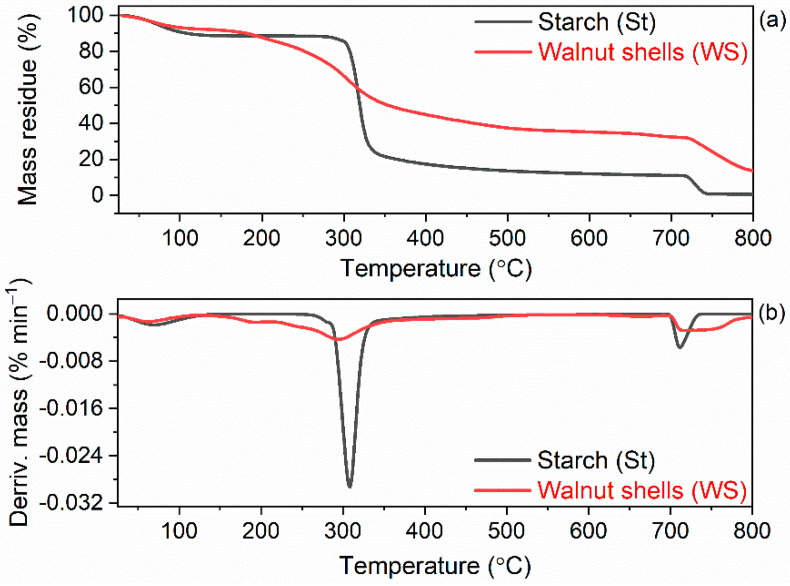
Thermogravimetric (TG) and derivative thermogravimetric (DTG) curves of starch and walnut shells (**a**) TG curves; (**b**) DTG curves.

In the first step of the research, thermogravimetric analysis (TG) was employed to determine the thermal stability of pure biofillers. The onset decomposition temperature (T_5%_) was established as the temperature at 5% mass loss in relation to the initial mass of the biofiller. TG and derivative thermogravimetric (DTG) curves for pure biofillers are presented in Figure 9, whereas results are summarized in Table 7.

The investigated fillers demonstrated a slightly different thermal behavior, which resulted from their different composition and nature (Figure 9). Thermal decomposition of starch was three-step process. The first stage of degradation proceeded in the temperature range of 25–150 °C, which corresponds to the water desorption [77]. The mass loss of dehydration stage, at which water bound within sample was evaporated, was approximately of 7.2%. The second stage of starch decomposition proceeded with the sharp degradation in the temperature range of 200–700 °C with the mass loss of 77.4%. At this temperature range, the degradation of starch organic components such as polysaccharides (amylose and amylopectin) occurred [78]. Moreover, analyzing the TG and DTG curves, it was observed that the main stage of starch degradation occurred at the temperature of 318 °C. Decomposition and dehydration have generally been considered as two independent processes associated with the degradation mechanisms of starch [78,79]. Next was the slow decomposition stage, which proceeded at the temperature above 700 °C, with the mass loss of 10.4%. This process is known as carbonization step, and the mass loss at this step was attributed to char as well as inorganic ash decomposition [77].

The thermal decomposition of ground walnut shells was also a three-step process. The first mass loss was 3.0% and occurred at a temperature of approximately 67 °C, so it resulted from desorption of water that was physically adsorbed by the walnut shells [80,81]. In the second step, the main degradation process occurred in the temperature range of 150–700 °C, with the mass loss of approximately 48.2%. The main peak on the DTG curve was determined at 305 °C. During the main degradation step of the walnut shells, decomposition of the cellulose, hemicellulose and a part of the lignin were proceeded [75,82,83]. The third mass loss of approximately 16.8% was observed at the temperature above 700 °C, which was due to a charring of the residue after thermal decomposition of the sample [84]. According to Uzun et al. [83], after 400 °C the passive pyrolysis zone of walnut shells starts and goes on up to 800 °C with a small mass loss of approximately 16%. The total mass loss resulting from walnut shells thermal decomposition was approximately 68%. The mass residue after thermal decomposition at 800 °C was approximately 32% and consisted mainly of char. The results obtained was in a good agreement with those reported by Xu et al. [85], who determined the residual mass after thermal decomposition of walnut shells to be 31.1%.

**Figure 10:**
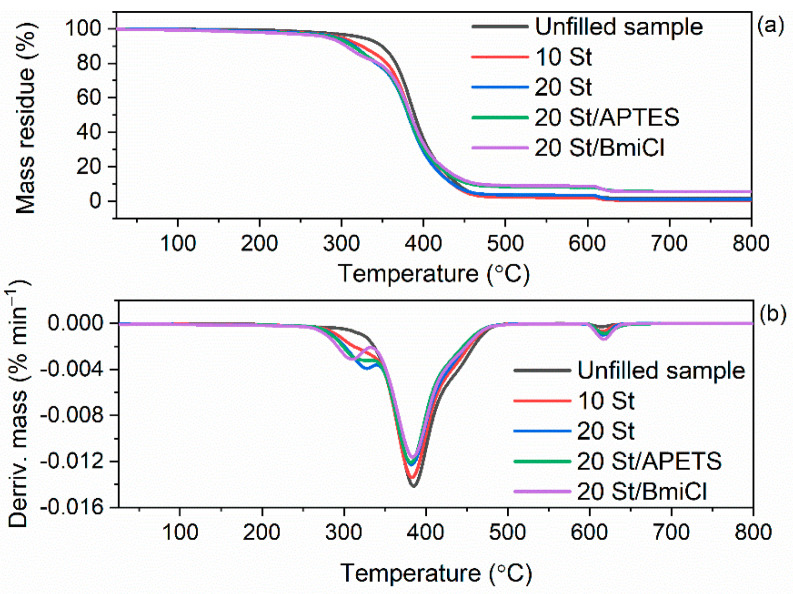
TG and DTG curves of the starch-filled NR vulcanizates containing BmiCl and APTES: (**a**) TG curves; (**b**) DTG curves.

**Figure 11:**
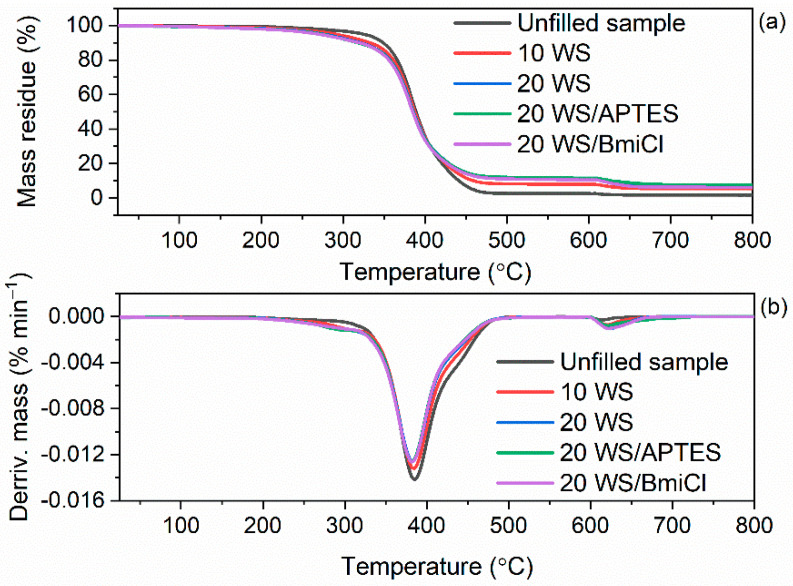
TG and DTG curves of the walnut shells-filled NR vulcanizates containing BmiCl and APTES: (**a**) TG curves; (**b**) DTG curves.

Having established the thermal stability of pure biofillers, i.e., starch and walnut shells, we then examined their influence on the thermal behavior of NR composites. The results of TG analysis of NR vulcanizates are summarized in Table 8, whereas TG and DTG curves are plotted in Figure 10 and Figure 11.

As expected, the type of biofiller and additives affected the onset decomposition temperature (T_5%_) of the NR vulcanizates, which was reduced as compared to the unfilled vulcanizate. In contrast, no influence of biofillers and additives on the T_DTG_, and therefore, the temperature of the maximum mass loss rate was achieved. T_DTG_ of the studied vulcanizates ranged from 399 °C to 401 °C. Thermal decomposition of the unfilled vulcanizate started at a temperature of approximately 322 °C and proceeded with the mass loss of 97.1%. Applying starch reduced the T_5%_ by 24 °C and 32 °C for 10 phr and 20 phr of this biofiller, respectively. Thus, the T_5%_ decreased with increasing amount of starch in the vulcanizate. Therefore, it was concluded that deterioration of the thermal stability of NR vulcanizates was due to the thermal decomposition of starch, which proceeded at a lower temperature than the thermal decomposition of NR in the unfilled vulcanizate. It is also confirmed by the small peak in the DTG curve, which was observed for the starch-filled vulcanizates at a temperature above 300 °C. It should be mentioned that thermal decomposition of pure starch proceeded with a sharp peak on the DTG curve at a temperature of approximately 318 °C. It resulted from the degradation of organic components of starch such as amylose and amylopectin [79].

Walnut shells reduced T_5%_ of NR vulcanizates by 33 °C (10 WS) and 48 °C (20 WS) compared to the unfilled benchmark. Thus, walnut shells deteriorated the thermal stability of NR vulcanizates to a greater extent than the starch. It was due to the lower thermal stability of pure walnut shells compared to the pure starch as confirmed by TG results of biofillers presented in Figure 9. The addition of BmiCl and APTES did not considerably affect the T_5%_ and T_DTG_ of NR vulcanizates. T_5%_ of the vulcanizates containing APTES and BmiCl was 3–10 °C lower compared to the vulcanizates without these additives.

The mass loss at 25–600 °C for the starch-filled vulcanizates was practically the same as for the unfilled benchmark, while it was slightly lower for the vulcanizates containing APTES and BmiCl (mass loss of approximately 91%). It resulted from the lower rubber content in the same amount of rubber compound compared to the unfilled vulcanizate.

Regarding the mass loss at 25–600 °C for the vulcanizates filled with walnut shells, it was approximately 5–8% lower compared to the unfilled benchmark and vulcanizates filled with starch. Ground walnut shells contain not only organic components, which decompose at this step, but also minerals, such as K, Ca and Mg, which do not degrade in the temperature range of 25–600 °C [54]. Therefore, the residue at 800 °C was significantly higher for the vulcanizates filled with walnut shells as compared to the unfilled one or those containing starch.

Although biofillers worsened the thermal stability compared to the unfilled vulcanizate, it should be noted that NR composites filled with starch or walnut shells were thermally stable up to a temperature of approximately 270 °C.

## 3. Materials and Methods

### 3.1. Materials

**Figure 12:**
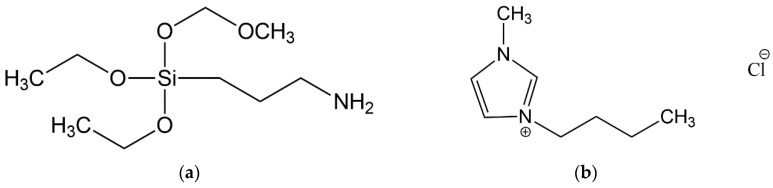
Structure of compounds used to improve the compatibility between elastomer matrix and biofillers: (**a**) (3-aminopropyl)-triethoxysilane (APTES); (**b**) 1-butyl-3-methylimidazolium chloride (BmiCl).

Natural rubber (NR, cis-1,4-polyisoprene) of RSS1 type with a density of 0.930–0.988 g/cm^3^ was supplied by Torimex Chemicals (Lodz, Poland). A conventional crosslinking system was used, containing sulfur with a purity of 99.9% (Siarkopol, Tarnobrzeg, Poland) as a curing agent, microsized zinc oxide (ZnO) with a specific surface area of 10 m^2^/g and purity of 99.0% (Huta Będzin, Będzin, Poland) as a vulcanization activator and 2-mercaptobenzothiazole (MBT) with purity of 98.0% applied as an accelerator (Brenntag Polska, Kedzierzyn-Kozle, Poland). Stearic acid (St.A.) purchased from Akzo Nobel (Amsterdam, The Netherlands) was used as the softener and filler-dispersing agent. Natural bioadditives with different characteristics such as ground walnut shells (WS) and corn starch (St) obtained from Plantago (Zlotow, Poland) were used as fillers for NR compounds. Additionally; ionic liquid, i.e., 1-butyl-3-methylimidazolium chloride (BmiCl) with purity of ≥99.0%, supplied by IoLiTec Ionic Liquids Technologies GmbH, Heilbronn, Germany; and aminosilane such as (3-aminopropyl)-triethoxysilane (APTES, Sigma–Aldrich, Schelldorf, Germany) were applied to improve the compatibility between the elastomer matrix and biocomponents added. The structure of BmiCl and APTES was presented in Figure 12.

### 3.2. Preparation and Characterization of NR Compounds

NR compounds were prepared according to the general formulations presented in Table 9, exploiting a laboratory two-roll mill (David Bridge & Co., Rochdale, UK, Country) with the following roll dimensions: D = 200 mm, L = 450 mm. The friction and the width of the gap between rollers were 1–1.2 and 1.5–3 mm, respectively, whereas the rotational speed of the front roll during compounding was 16 min^−1^. The average temperature of the rolls during rubber preparation was approximately 30 °C. In the case of NR compounds containing ionic liquid and silane, biofillers were mixed with APTES and BmiCl, respectively, before being incorporated into the rubber compounds. Then, the mixtures of biofillers with a proper additive were introduced into the rubber composites.

In order to obtain plates of the vulcanizates, NR compounds were cured at 160 °C, at 15 MPa pressure using the optimal vulcanization times determined during rheometric tests. The vulcanization of NR compounds was carried out using a hydraulic press with electrical heating.

The curing behavior of NR compounds was investigated at 160 °C according to the ISO 6502 [86] standard procedures. The rotorless D-RPA 3000 rheometer (MonTech, Buchen, Germany) was employed to perform measurements. Rheometric tests were carried out for specimens with a mass of approximately 7 g. The optimal vulcanization time (t_90_) was determined as a parameter corresponding to the time required for the rubber compound to reach 90% of the maximum torque as given by Equation (1), where Δ*S* is the torque increase during rheometric test, calculated as the difference between the maximum (S_max_) and the minimum (S_min_) torque. Adapting the similar equation, the scorch time (t_02_) was determined.

(1) $$S_{90}=0.90\Delta S+S_{\min}$$

To determine the effect of biofillers, BmiCl and APTES on the temperatures and enthalpy of NR vulcanization, a differential scanning calorimeter DSC1 (Mettler Toledo, Greifensee, Switzerland) was employed. Using a STARe software (Version 16.4, 2022, Greifensee, Switzerland), the onset temperature of the peak corresponding to the crosslinking reactions was determined according to the ISO 11357-1 [87] standard. The measurements were carried out for small pieces of rubber compounds with a mass of approximately 10 mg, which were placed in a hermetically sealed aluminum crucible with a capacity of 40 µL and heated from −100 °C to 250 °C, with a constant heating rate of 10 K/min. In addition, the glass transition temperature of the elastomer was determined during DSC measurements according to ISO 11357-2 [88]. Liquid nitrogen was used for cooling the sample before the measurement, whereas the nitrogen (flow rate 80 mL/min) was applied as the protective gas.

According to the ISO 1817 [89] standard and using the Flory–Rehner equation [90], the density of crosslinks in the cured elastomer network was examined by the equilibrium swelling of vulcanizates in toluene. Small pieces of the vulcanizates with masses in the range of 20–30 mg were swollen in toluene for 48 h at room temperature. Then, after removing the solvent and weighing the samples, they were dried at 50 °C for another 48 h. At the end, the dried samples were reweighed. The crosslink density was calculated using the Huggins parameter of NR-toluene interaction (*χ*) given by Equation (2), where *V_r_* is the volume of the elastomer fraction in swollen gel [41].

(2) $$\chi =0.780+0.404V_{r},$$

Scanning electron microscopy (SEM) was employed to study the dispersion of biofillers and other additives in the NR elastomer matrix. SEM images of NR vulcanizates were taken using a LEO1450 SEM microscope (Carl Zeiss AG, Oberkochen, Germany). Prior to the measurement, vulcanizates were broken down using liquid nitrogen. In the next step, their fractures were coated with carbon and then examined. Energy-dispersive X-ray spectroscopy (EDS) was employed to investigate the morphology and size of corn starch and ground walnut shells particles. Samples of pure fillers were coated with carbon to improve the quality of the obtained results. EDS spectra were collected to establish the elemental composition of the fillers used.

Mechanical properties of NR vulcanizates under static conditions were investigated following to the ISO 37 [91] standard procedure using Zwick Roell 1435 (Ulm, Germany) universal testing machine. Tensile tests were carried out for six dumbbell-shaped samples of each vulcanizate with a thickness of approximately 2 mm and the width of the measuring section of 4 mm. The crosshead speed during tensile tests was 500 mm/min.

Zwick Roell 3105 (Ulm, Germany) hardness tester was used to establish the hardness of NR vulcanizates using Shore’s A method according to the standard ISO 868 [92]. Measurements were performed for disc-shaped specimens.

Dynamic mechanical analysis (DMA) was performed in tension mode using a DMA/SDTA861e analyzer (Mettler Toledo, Greifensee, Switzerland). The measurements were carried out in the temperature range of −100–80 °C with a heating rate of 3 K/min, a frequency of 1 Hz and a strain amplitude of 4 µm. Tests were performed for strip-shaped specimens with a width of 4 mm, length of 10.5 mm and a thickness of approximately 2 mm. Liquid nitrogen was applied as a cooling medium. The temperature of the elastomer glass transition (T_g_) was designated from the maximum of the tan δ = f (T) curve, where tan δ is the mechanical loss factor, and T is the measurement temperature.

Thermogravimetric (TG) analysis was carried out to study the thermal behavior of pure biofillers and consequently their influence on the thermal stability of NR vulcanizates. Thermogravimetry/differential scanning calorimetry TGA/DSC1 analyzer (Mettler Toledo, Greifensee, Switzerland) was applied to perform measurements for the samples with a mass of approximately 10 mg. Samples were placed in the open alumina crucibles with a capacity of 70 µL. Measurements were carried out using two-step procedure. First, specimens were heated in the temperature range of 25–600 °C in an argon atmosphere (gas flow of 50 mL/min) with a heating rate of 20 K/min. Subsequently, the gas was changed into air (gas flow of 50 mL/min), and heating was continued up to 800 °C with the same heating rate. In the case of pure biofillers, the powders were heated in the temperature range of 25–700 °C in an argon atmosphere, and then the gas was changed into air. The heating was continued up to 800 °C with the same heating rate (20 K/min).

Following to the ISO 188 standard [93], the thermo-oxidative aging of the vulcanizates was investigated. Plates of NR vulcanizates with a thickness of approximately 2 mm were stored in a drying chamber (Binder, Tuttlingen, Germany) at 70 °C for ten days (240 h). To determine the resistance of the vulcanizates to prolonged thermo-oxidation, their crosslink densities, mechanical properties and hardness were established and compared with the values obtained for nonaged vulcanizates. The aging coefficient (A_f_), which quantifies the resistance of material to prolonged thermo-oxidation, was calculated according to Equation (3) [94], where TS is the tensile strength of vulcanizates, and *E_b_* is the elongation at break.

(3) $$A_{f}=\frac{{\left(E_{b}\times TS\right)}_{after\;aging}}{{\left(E_{b}\times TS\right)}_{before\;aging}},$$

## 4. Conclusions

The aim of this work was to study the influence of biofillers on the curing characteristics and performance of natural rubber biocomposites. Corn starch and ground walnut shells were used as biodegradable and eco-friendly fillers of NR composites.

The performed research proved that starch and ground walnut shells can be classified as inactive fillers, which can be used alternatively to commercial inactive fillers, e.g., chalk or talc. Furthermore, ionic liquids, i.e., BmiCl, and aminosilane, i.e., APTES, can be successfully used to support the vulcanization and improve the performance of NR composites filled with starch and walnut shells.

Applied biofillers affected the rheometric properties and cure characteristics of NR compounds. Both biofillers and additives used caused an increase in the maximum torque during vulcanization due to the introduction of the rigid phase of the filler into the elastomer matrix. The improvement of torque increase indicated the development of spatial structure in the crosslinked elastomer network, which was confirmed by the enhancement of the crosslink density of vulcanizates, especially those containing APTES and BmiCl as compared to the unfilled sample. These additives not only improved the crosslink density but also had a beneficial effect on the optimal vulcanization time and the onset vulcanization temperature, which were reduced compared to NR compounds without APTES and BmiCl.

Vulcanizates containing starch, especially with addition of APTES and BmiCl, exhibited improved tensile properties as compared to the unfilled benchmark. It resulted from the higher crosslink density of these vulcanizates and from the homogenous dispersion of starch, which was mainly due to the acting of BmiCl. Regardless of the biofiller and additives used, NR vulcanizates exhibited approximately 200–300% lower elongation at break than the unfilled benchmark. It resulted from the increased crosslink density of these vulcanizates and increased stiffness due to the introduction of biofillers into the elastomer matrix. In contrast, biofillers APTES and BmiCl did not significantly affect the ability of NR vulcanizates to dampen vibrations.

Walnut shell-filled vulcanizates demonstrated improved resistance to thermo-oxidative aging as compared to the unfilled benchmark and starch-filled composites. It resulted from the presence of lignin in the composition of walnut shells, the components of which belong to polyphenols, which have an antioxidant activity. The beneficial influence of BmiCl on the resistance to prolonged thermo-oxidation of the starch-filled NR composites was observed.

## Figures and Tables

**Table 1:** Cure characteristics at 160 °C of NR compounds and crosslink density of vulcanizates containing biofillers (S_min_, minimum torque; S_max_, maximum torque; ∆S, torque increase; t_02_, scorch time; t_90_, optimal vulcanization time; standard deviations: S_min_ ± 0.2 dNm, S_max_ ± 0.8 dNm, ΔS ± 0.8 dNm, t_02_ ± 0.5 min, t_90_ ± 0.5 min; ν_t_ ± 0.2 × 10^−5^ mole/cm^3^).

Compounds	S_min_ (dNm)	S_max_ (dNm)	∆S (dNm)	t_02_ (min)	t_90_ (min)	$\nu_{t}\times 10^{-5}\;\;(\mathrm{mole}/{\mathrm{cm}}^{3})$
Unfilled sample	0.5	5.4	4.9	2	7	1.2
NR compounds filled with starch						
10 St	0.4	6.2	5.9	3	5	2.8
20 St	0.4	7.2	6.8	3	5	2.9
20 St/APTES	0.6	7.8	7.2	1	3	3.3
20 St/BmiCl	0.2	8.5	8.3	1	3	3.4
NR compounds filled with walnut shells						
10 WS	0.4	6.1	5.6	3	5	2.6
20 WS	0.3	6.2	5.9	3	4	2.1
20 WS/APTES	0.4	6.8	6.4	1	3	2.6
20 WS/BmiCl	0.2	7.5	7.3	1	3	2.8

**Table 2:** Differential scanning calorimetry (DSC) of NR compounds containing biofillers (T_g_, glass transition temperature; ∆C_p_, heat capacity; ∆H, enthalpy of crosslinking; T_g_ ± 1 °C, ∆C_p_ ± 0.1 J/g × K, temperature ± 7.0 °C; ΔH ± 1.3 J/g).

Compounds	T_g_ (°C)	∆C_p_ (J/g × K)	Temperature of Crosslinking (°C)	∆H (J/g)
Unfilled sample	−62	0.43	167–227	15.6
NR compounds filled with starch				
10 St	−62	0.42	165–220	12.5
20 St	−62	0.49	164–220	11.7
20 St/APTES	−62	0.43	133–221	17.6
20 St/BmiCl	−62	0.43	130–224	18.4
NR compounds filled with walnut shells				
10 WS	−62	0.41	168–220	9.9
20 WS	−62	0.41	165–220	10.5
20 WS/APTES	−62	0.43	131–208	11.5
20 WS/BmiCl	−62	0.43	131–209	12.7

**Table 3:** EDS analysis of pure biofillers (wt. %, weight percentage).

Biofiller	Wt. (%)													
	C K	O K	Al K	Na K	Mg K	Si K	P K	S K	Cl K	K K	Ca K	Fe K	Cu K	Total
Starch	49.55	50.00	0.45	-	-	-	-	-	-	-	-	-	-	100
Walnut shells	46.24	42.72	-	0.01	0.15	0.22	0.15	0.27	0.22	8.13	1.41	0.17	0.31	100

**Table 4:** Mechanical properties and hardness of NR vulcanizates containing biofillers (SE_300_, stress at 300% relative elongation; TS, tensile strength; EB, elongation at break; H, hardness).

NR Vulcanizates	SE_300_ (MPa)	TS (MPa)	EB (%)	H (Shore A)
Unfilled sample	1.2 ± 0.1	10.4 ± 0.1	820 ± 20	31 ± 1
NR vulcanizates filled with starch				
10 St	1.6 ± 0.1	12.1 ± 0.2	607 ± 22	33 ± 1
20 St	1.6 ± 0.1	11.7 ± 0.3	673 ± 16	33 ± 1
20 St/APTES	2.2 ± 0.1	11.0 ± 0.5	570 ± 26	35 ± 1
20 St/BmiCl	2.2 ± 0.1	11.5 ± 0.4	507 ± 9	38 ± 1
NR vulcanizates filled with walnut shells				
10 WS	1.4 ± 0.1	11.3 ± 0.3	668 ± 21	34 ± 1
20 WS	1.3 ± 0.1	10.8 ± 0.5	686 ± 21	32 ± 1
20 WS/APTES	1.7 ± 0.1	9.9 ± 0.6	645 ± 8	32 ± 1
20 WS/BmiCl	1.8 ± 0.1	11.1 ± 0.5	588 ± 28	34 ± 1

**Table 5:** Glass transition temperature (T_g_) determined by dynamic mechanical analysis (DMA) and mechanical loss factor (tan δ) of NR vulcanizates containing biofillers.

NR Vulcanizates	T_g_ (°C)	Tan δ_Tg_ (-)	Tan δ_25 °C_ (-)	Tan δ_50 °C_ (-)
Unfilled sample	−68 ± 1	2.6 ± 0.1	0.06 ± 0.02	0.05 ± 0.02
NR vulcanizates filled with starch				
NR/10 St	−68 ± 1	2.5 ± 0.1	0.05 ± 0.02	0.05 ± 0.02
NR/20 St	−68 ± 1	2.4 ± 0.1	0.06 ± 0.02	0.05 ± 0.02
NR/20 St/APTES	−68 ± 1	2.4 ± 0.1	0.07 ± 0.02	0.05 ± 0.02
NR/20 St/BmiCl	−69 ± 1	2.5 ± 0.1	0.05 ± 0.02	0.04 ± 0.02
NR vulcanizates filled with walnut shells				
NR/10 WS	−68 ± 1	2.4 ± 0.1	0.07 ± 0.02	0.05 ± 0.02
NR/20 WS	−69 ± 1	2.3 ± 0.1	0.07 ± 0.02	0.07 ± 0.02
NR/20 WS/APTES	−69 ± 1	2.5 ± 0.1	0.08 ± 0.02	0.05 ± 0.02
NR/20 WS/BmiCl	−70 ± 1	2.4 ± 0.1	0.08 ± 0.02	0.06 ± 0.02

**Table 6:** Thermo-oxidative aging coefficient (A_f_) of NR vulcanizates filled with starch and walnut shells.

NR Vulcanizates	A_f_ (-)
Unfilled sample	0.6 ± 0.1
NR vulcanizates filled with starch	
10 St	0.6 ± 0.1
20 St	0.6 ± 0.1
20 St/APTES	0.7 ± 0.1
20 St/BmiCl	0.8 ± 0.1
NR vulcanizates filled with walnut shells	
10 WS	0.8 ± 0.1
20 WS	0.8 ± 0.1
20 WS/APTES	0.7 ± 0.1
20 WS/BmiCl	0.8 ± 0.1

**Table 7:** Onset temperature of thermal decomposition (T_5%_), DTG peak temperature (T_DTG_), and total mass loss (∆m) during decomposition of pure starch and walnut shells (SD: T_5%_ ± 1.2 °C; T_DTG_ ± 1.3 °C; ∆m ± 1.3%).

Biofiller	T_5%_ (°C)	T_DTG_ (°C)	∆m_25–150 °C_ (%)	∆m_150–700 °C_ (%)	∆m_700–800 °C_ (%)	Residue at 800 °C (%)
Starch	75	318	7.2	77.4	10.4	5.0
Walnut shells	78	305	3.0	48.2	16.8	32.0

**Table 8:** Onset temperature of thermal decomposition (T_5%_), DTG peak temperature (T_DTG_), and total mass loss (∆m) during decomposition of NR composites filled with starch and walnut shells (SD: T_5%_ ± 1.1 °C; T_DTG_ ± 1.3 °C; ∆m ± 1.3%).

NR Vulcanizates	T_5%_ (°C)	T_DTG_ (°C)	∆m_25–600 °C_ (%)	∆m_600–800 °C_ (%)	Residue at 800 °C (%)
Unfilled sample	322	401	97.1	0.9	1.9
NR vulcanizates filled with starch					
10 St	298	400	97.1	1.6	1.3
20 St	290	399	96.2	2.3	1.5
20 St/APTES	285	399	91.2	2.9	6.9
20 St/BmiCl	280	401	91.4	3.2	5.4
NR vulcanizates filled with walnut shells					
10 WS	289	401	91.7	2.4	5.9
20 WS	274	400	89.0	3.7	7.3
20 WS/APTES	271	399	87.9	4.0	8.1
20 WS/BmiCl	268	400	88.0	4.3	7.7

**Table 9:** General recipes of natural rubber (NR) compounds, parts per hundred of rubber (phr); (MBT, 2-mercaptobenzothiazole; St.A., stearic acid; St, starch; WS, walnut shells; BmiCl, 1-butyl-3-methylimidazolium chloride; APTES, (3-aminopropyl)-triethoxysilane).

Ingredient, Phr	Unfilled Sample (0)	NR/St (1–2)	NR/St/BmiCl (3)	NR/St/APTES (4)	NR/WS (5–6)	NR/WS/BmiCl (7)	NR/WS/APTES (8)
NR	100	100	100	100	100	100	100
Sulfur	2	2	2	2	2	2	2
ZnO	5	5	5	5	5	5	5
MBT	2	2	2	2	2	2	2
St.A.	1	1	1	1	1	1	1
St	-	10; 20	20	20	-	-	-
WS	-	-	-	-	10; 20	20	20
BmiCl	-	-	2	-	-	2	-
APTES	-	-	-	2	-	-	2

## Data Availability

The data presented in this study are available on request from the corresponding author.
